# NgsRelate: a software tool for estimating pairwise relatedness from next-generation sequencing data

**DOI:** 10.1093/bioinformatics/btv509

**Published:** 2015-08-30

**Authors:** Thorfinn Sand Korneliussen, Ida Moltke

**Affiliations:** ^1^Center for GeoGenetics and; ^2^Department of Biology, University of Copenhagen, 2200 Copenhagen, Denmark

## Abstract

**Motivation**: Pairwise relatedness estimation is important in many contexts such as disease mapping and population genetics. However, all existing estimation methods are based on called genotypes, which is not ideal for next-generation sequencing (NGS) data of low depth from which genotypes cannot be called with high certainty.

**Results**: We present a software tool, NgsRelate, for estimating pairwise relatedness from NGS data. It provides maximum likelihood estimates that are based on genotype likelihoods instead of genotypes and thereby takes the inherent uncertainty of the genotypes into account. Using both simulated and real data, we show that NgsRelate provides markedly better estimates for low-depth NGS data than two state-of-the-art genotype-based methods.

**Availability**: NgsRelate is implemented in C++ and is available under the GNU license at www.popgen.dk/software.

**Contact**: ida@binf.ku.dk

**Supplementary information:** Supplementary data are available at *Bioinformatics* online.

## 1 Introduction

Estimation of how related two individuals are from genetic data plays a key role in several research areas, including medical genetics and population genetics. For example, in medical genetics it is used for excluding closely related individuals from association studies and thereby to avoid inflated false positive rates. How related two individuals are is usually described through the concept of identity-by-descent (IBD), i.e. genetic identity due to a recent common ancestor. Historically, several summary statistics have been used, such as the kinship coefficient θ, however almost all of these statistics can be calculated from $R=(k_{0},k_{1},k_{2})$, where *k_m_* is the fraction of genome in which the two individuals share *m* alleles IBD. For example $\theta =\frac{k_{1}}{4}+\frac{k_{2}}{2}$. We will therefore here focus on *R.*

Many estimators for *R* have been proposed, both method of moments (Purcell *et al.*, 2007; Ritland, 1996) and maximum likelihood (ML) estimators (Thompson, 1975). Common to them all is that they are based on genotype data and it has been shown that they work well on single nucleotide polymorphism (SNP) chip data. However, next-generation sequencing (NGS) is becoming increasingly common and often NGS data are only of low depth, which means that genotypes can only be called with high uncertainty (O’Rawe *et al.*, 2015). For such data it has been shown that it can be an advantage to take the uncertainty of the genotypes into account by basing statistical methods on so-called genotype likelihoods (GLs), instead of genotypes (Skotte *et al.*, 2013). Motivated by this we developed NgsRelate; a ML method for estimating the pairwise relatedness parameter *R* from NGS data based on GLs. In the following, we present this method and show that for low-depth NGS data it performs markedly better than two state-of-the-art genotype-based methods.

## 2 Methods

To estimate *R* for two non-inbred individuals *i* and *j* we use the following probabilistic framework: Let *D^i^*^ ^= $\left(D_{1}^{i},D_{2}^{i},\ldots ,D_{L}^{i}\right)$ and *D^j^*^ ^= $\left(D_{1}^{j},D_{2}^{j},\ldots ,D_{L}^{j}\right)$ denote the observed NGS data for *i* and *j* at *L* diallelic loci and *G^i^*^ ^= $\left(G_{1}^{i},G_{2}^{i},\ldots ,G_{L}^{i}\right)$ and *G^j^*^ ^= $\left(G_{1}^{j},G_{2}^{j},\ldots ,G_{L}^{j}\right)$ denote the true unobserved genotypes at the *L* loci. Further, let $X_{l}\in \{0,1,2\}$ denote the unobserved number of alleles *i* and *j* share IBD at locus *l.* Finally, let the two alleles at each locus be denoted *A* and *a* and the frequencies of the *A* alleles be denoted *f^A^^ ^= *$\left(f_{1}^{A},f_{2}^{A},\ldots ,f_{L}^{A}\right)$. Then, assuming the loci are independent and that *f^A^* is known the likelihood function for *R*, can be written:

$$L(R|D^{i},D^{j},f^{A})=\prod_{l=1}^{L}\sum_{m\in \{0,1,2\}}P(D_{l}^{i},D_{l}^{j}|X_{l}=m,f_{l}^{A})P(X_{l}=m|R)$$

with $P(X_{l}=m|R)=k_{m}$ and

$$\begin{array}{l} P(D_{l}^{i},D_{l}^{j}|X_{l}=m,f_{l}^{A})\\ =\sum_{G_{l}^{i},G_{l}^{j}\in {\left\{0,1,2\right\}}^{2}}P(D_{l}^{i}|G_{l}^{i})P(D_{l}^{j}|G_{l}^{j})P(G_{l}^{i}|f_{l}^{A})P(G_{l}^{j}|f_{l}^{A},X_{l}=m,G_{l}^{i}) \end{array}$$

Here $P(D_{l}^{i}|G_{l}^{i})$ and $P(D_{l}^{j}|G_{l}^{j})$ are GLs, which can be estimated using ANGSD (Korneliussen *et al.*, 2014) and $P(G_{l}^{i}|f_{l}^{A})$ and $P(G_{l}^{j}|f_{l}^{A},X_{l}$ = $m,G_{l}^{i})$ are given in Supplementary Table S1–S2. *f^A^* and major and minor alleles can be precalculated from NGS data using ANGSD or from SNP chip data. NgsRelate provides ML estimates of *R* by finding the value of *R* that maximizes this likelihood function with an Expectation Maximization algorithm (Supplementary Data). Like all other ML estimators, this estimator is consistent and we note that this is also true if the assumption of independence between loci is violated, since the function that is optimized then becomes a composite likelihood function. We also note that if the genotypes are known with certainty the GLs will be 0 for all but the true genotype and in that case the method reduces to the ML method in Choi *et* *al.* (2009). In all other cases the uncertainty is taken into account by summing over all possible true genotypes and weighing each according to their GLs.

## 3 Results and discussion

**Fig. 1.**
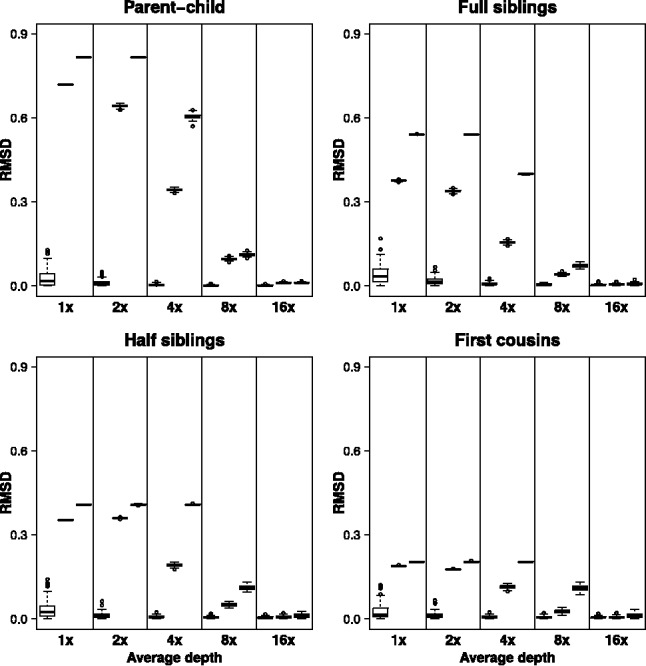
Root mean square deviation (RMSD) between estimated and simulated *R* for 100 of each combination of four relationship types and five average sequencing depths 1, 2, 4, 8 and 16 (see Supplementary Fig. S5 for results for unrelated pairs). For each combination estimates were obtained with NgsRelate (left), genotype-based ML (middle) and PLINK (right). RMSD will be zero if the estimate is equal to the simulated *R*

To test NgsRelate we used both simulated and real data. We first simulated NGS data for 100 000 diallelic loci from 100 pairs of individuals from each of the relationships: parent–child, full siblings, half-siblings, first cousins and unrelated individuals. To make it possible to assess how NgsRelate’s performance depends on average sequencing depth we simulated such data for five different average depths ranging from low (1, 2 and 4×) over medium (8×) to relatively high depth (16×). From the simulated data we calculated GLs, which we applied NgsRelate to. We also called genotypes based on the maximum GLs and applied the genotype-based ML method from Choi *et* *al.* (2009) and PLINK (Purcell *et al.*, 2007) to these called genotypes. See Supplementary Data for details. The simulations showed that all three methods perform well on high-depth data, but that the two genotype-based methods did not provide accurate estimates of *R* for the related pairs based on low- and medium-depth data (Fig. 1). Further inspection of the results revealed that for all the related pairs these two methods tend to overestimate *k*_0_ and thereby make the pairs look less related (Supplementary Figs S1–S5). NgsRelate on the other hand performs well on medium and low-depth data down to 4× (Fig. 1). Even for 2× data it is only slightly biased (Supplementary Figs S1–S5) and for 1× it has large variance, yet it still performs markedly better than the other two methods (Fig. 1). Hence, the simulations suggest that for low-depth NGS data NgsRelate outperforms the two genotype-based methods.

**Fig. 2.**
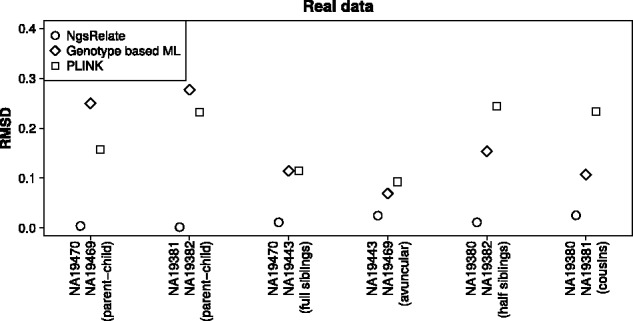
RMSD between the estimated and the true *R* for six pairs of ∼4× genomes. RMSD will be 0 if the estimate is equal to the true *R*

To assess if this holds true for real data we then applied the three methods to low-depth (∼4×) NGS data from six genomes from the 1000 Genomes Project Consortium (2012). These individuals have also been SNP chip genotyped (International HapMap 3 Consortium, 2010), and six of the pairs have been reported to be related. We applied NgsRelate to GLs calculated from the low-depth NGS data using ANGSD and applied the two other methods to genotypes called from these GLs. To limit the amount of genotype calling errors only data from sites with depth above 2 in both genomes and a minor allele frequency above 0.05 were included in the genotype-based analyses. Next, we estimated *R* from the high-quality SNP chip genotypes using a state-of-the-art genotype-based method to achieve accurate estimates of *R*, which we used as a proxy for the true values when assessing the NGS data-based estimates. For all six-related pairs the estimates from NgsRelate differed markedly less from the ‘true’ values (Fig. 2 and Supplementary Fig. S6), e.g. the difference in *k*_0_ ranged from 0.002 to 0.031 for NGSrelate, whereas they ranged from 0.081 to 0.31 for genotype-based ML estimator and from 0.096 to 0.25 for PLINK. In all cases *k*_0_ was overestimated, though, note that the opposite was observed for PLINK when we changed the quality filtering of the genotypes (Supplementary Data), suggesting that estimates from the genotype-based methods depend highly on filtering choices. However, all the real data results supported the conclusion from the simulations: for low-depth NGS data NgsRelate provides more accurate estimates.

## Supplementary Material

Supplementary Data
