# Supplementary material for: NgsRelate: a software tool for estimating pairwise relatedness from next-generation sequencing data
Source: Bioinformatics. 2015 Aug 30;31(24):4009–11. doi: 10.1093/bioinformatics/btv509 (PMC4673978; doi:10.1093/bioinformatics/btv509)
Supplement: Supplementary Data [file supp_31_24_4009__index.html]

NgsRelate: a software tool for estimating pairwise relatedness from next-generation sequencing data — NgsRelate: a software tool for estimating pairwise relatedness from next-generation sequencing data — Supplementary Data 

# NgsRelate: a software tool for estimating pairwise relatedness from next-generation sequencing data

## Supplementary Data

files

- Supplementary Data - pdf file
