# Supplementary material for: NgsRelate: a software tool for estimating pairwise relatedness from next-generation sequencing data
Source: Bioinformatics. 2015 Aug 30;31(24):4009–11. doi: 10.1093/bioinformatics/btv509 (PMC4673978; doi:10.1093/bioinformatics/btv509)
Supplement: Supplementary Data [file supp_btv509_KorneliussenAndMoltkeSupplementaryData_v4.pdf]

Supplementary Data for *NgsRelate: a software tool for estimating pairwise relatedness from next-generation sequencing data*

Thorfinn Sand Korneliussen and Ida Moltke

Supplementary Text

The EM-algorithm

For performing the maximum likelihood estimation we use an EM-algorithm very similar to the one presented in Choi *et al.* (2009): For $n = 1$ we set the values of $k_0^{(n)}$, $k_1^{(n)}$ and $k_2^{(n)}$ to random non-negative numbers that sum to 1. Then for $n = 2, 3, ...$ we iteratively update these value to:

$$k_m^{(n)} = \frac{1}{L}\sum_{l=1}^{L} P(X_l = m|D_l^i, D_l^i, k_0^{(n-1)}, k_1^{(n-1)}, k_2^{(n-1)})$$
$$= \frac{1}{L}\sum_{l=1}^{L} \frac{k_m^{(n-1)} P(D_l^i, D_l^i|X_l = m)}{\sum_{m_*=0}^{2} k_{m_*}^{(n-1)} P(D_l^i, D_l^i|X_l = m_*)}$$

where for any m (or m_*)

$$P(D_l^i, D_l^j|X_l = m)$$
$$= \sum_{G_l^i, G_l^j \in \{0,1,2\}^2} P(D_l^i|G_l^i)P(D_l^j|G_l^j)P(G_l^i|f_l^A)P(G_l^j|f_l^A, X_l = m, G_l^i)$$

We stop updating when the likelihood for $(k_0^{(n)}, k_1^{(n)}, k_2^{(n)})$ and $(k_0^{(n-1)}, k_1^{(n-1)}, k_2^{(n-1)})$ differ by less than 1×10^{-6}. To make the algorithm faster we accelerated it using the squared iterative method S3 (Varadhan and Roland, 2008).

Simulation of NGS data

We simulated NGS data for 100 pairs of individuals from each of the five relationship types: parent-child (i.e. $E((k_0, k_1, k_2)) = (0, 1, 0)$), full siblings (i.e. $E((k_0, k_1, k_2)) = (0.25, 0.5, 0.25)$), half siblings (i.e. $E((k_0, k_1, k_2)) = (0.5, 0.5, 0)$), first cousins (i.e. $E((k_0, k_1, k_2)) = (0.75, 0.25, 0)$) and unrelated individuals (i.e.$E((k_0, k_1, k_2)) = (1, 0, 0)$). For each pair of individuals of a given relationship type we simulated data with an average depth of each of the values 1,2,4,8 and 16 for 100,000 di-allelic loci independently as follows: For each locus l we first sampled an allele frequency from a uniform distribution on $[0.05, 0.95]$ and an IBD state from a multinomial distribution with support $\{0, 1, 2\}$ and probability vector $E((k_0, k_1, k_2))$. Then we sampled genotypes for the two individuals at l based on the sampled allele frequency and IBD state assuming Hardy-Weinberg Equilibrium (HWE). Finally, for each of the two individuals, i, we sampled the sequencing depth d at locus l from a poisson distribution with mean equal to the relevant average depth and sampled d bases $D_i^l = (b_1, ..., b_d)$ according to the individual's genotype, G, at locus l with a per base probability of a sequencing error of P. We used P=0.005 and in the case of sequencing error we replaced the sampled base with the other base known to be present in the locus. In other words, for each individual we sampled d bases of type A or a, each according to the distribution

$$P(b_w = a|G) = \begin{cases} 0.995 & G = aa \\ 0.5 & G = Aa \\ 0.005 & G = AA \end{cases}$$

Population allele frequencies are never known with full certainty. They will usually be estimated from a reference panel e.g. a SNP chip dataset. Therefore, we did not use the "true" allele frequencies simulated above when estimating relatedness from the simulated data. Instead we mimicked the uncertainty in the allele frequencies by simulating genotype data for 99 unrelated individuals for each simulated pair of related individuals using the "true" allele frequencies and assuming HWE. Then we based all the relatedness estimates on allele frequencies estimated from these simulated genotypes combined with the genotypes of the pair of related individuals.

Calculating genotype likelihoods and calling genotypes for simulated data

We calculated genotype likelihoods (GLs) from the simulated NGS data by using the model from McKenna *et al.* (2010) with the error parameter P set to 0.005. That is, for each of the three possible genotypes, $G = A_1A_2$, at locus l we calculated the likelihood of observing the NGS data $D_l^i = (b_1, ..., b_d)$ consisting of d observed bases from individual i at locus l as

$$L(G = A_1A_2|D_l^i) = \prod_{w=1}^{d} (\frac{1}{2}P(b_w|A_1) + \frac{1}{2}P(b_w|A_2))$$

with

$$P(b_w|A) = \begin{cases} P & b_w \neq A \\ 1-P & b_w = A \end{cases}$$

We called the genotype for individual i at locus l by calling the genotype with the highest GL. Note that as d increases, the chance that the true genotype will have the highest GL increases, leading to a correct genotype call.

Detailed description of the analyses of real data

We downloaded bam files with low-depth NGS data (∼4x) for six individuals from the 1000 genomes project webpage (1000 Genomes Project Consortium *et al.*, 2012): NA19380, NA19381, NA19382, NA19470, NA19469 and NA19443, all from Luhya in Webuye, Kenya (LWK). We also downloaded the HapMap 3 LWK SNP chip data (International HapMap 3 Consortium *et al.*, 2010), which includes the same six samples as well as 84 other LWK samples. From the bam files we calculated GLs for all the diallelic sites in the SNP chip dataset with minor allele frequency above 0.05 using the SAMtools genotype likelihood model (Li , 2010) as implemented in ANGSD (Korneliussen *et al.*, 2014). Only reads with mapping quality score above 30 and bases with a q-score above 20 were used and only GLs for the 3 possible genoypes (based on knowledge from the SNP chip data) were calculated. NgsRelate was run on these GLs as and allele frequencies estimated from the SNP chip data. To be able to assess the correctness of the estimates we also estimated R for these six pairs from the SNP chip data using PLINK (Purcell *et al.*, 2007). Finally, we called genotypes from the GLs using the same approach as we did for the simulated data and then applied PLINK and the ML methods from Choi *et al.* (2009) to the called genotypes. All the results can be seen in Fig. S6 and Table S3.

Choice of genotype likelihood calculation method

To assess the impact of the choice of genotype likelihood model may have on NgsRelate estimates, we repeated the NgsRelate analysis of the real data with genotype likelihoods calculated using GATK instead SAMtools. The resulting estimates were almost identical to the estimates reported in Table S3. The largest difference was less than 0.005, suggesting that the choice of genotype likelihood model has a little impact on the NgsRelate method.

Impact of quality filtering on genotype based methods

To assess the impact of the choice of quality filters, we reran the PLINK analyses of the real data after applying an additional quality filter. Specifically, we discarded all sites where the posterior probability of the called genotype was less than 0.95. The posterior probabilities were calculated from the genotype likelihoods assuming a uniform prior. This additional PLINK analysis gave different estimates than the one shown in Table S3, e.g. for the 6 related pairs the new PLINK estimates of k_0 were up to 0.12 lower than the SNP chip based estimates, as opposed to up to 0.25 higher as in Table S3. These results suggest that genotype based relatedness estimates from NGS data

can be sensitive to choice of quality filtering. However, for both the filtering choices investigated in this study, NgsRelate gave more accurate relatedness estimates than the genotype based methods. For estimates based on quality filtered data this was particularly the case for the unrelated pairs among the 6 individuals, where PLINK underestimated k_0 by between 0.11 and 0.35. In comparison NgsRelate underestimated k_0 by at most 0.02.

Calculation of RMSD

We calculated RMSD between the true value of R, $R^{true} = k_0^{true}, k_1^{true}, k_2^{true})$, and an a given estimate of R, $R^{est} = (k_0^{est}, k_1^{est}, k_2^{est})$ using the following formula:

$$\sqrt{\frac{1}{3}((k_0^{true} - k_0^{est})^2 + (k_1^{true} - k_1^{est})^2 + (k_2^{true} - k_2^{est})^2)}$$

Computational time

The NgsRelate analyses of all 15 pairs among the 6 real data samples took ∼36 seconds with a 2.9Ghz CPU.

Acknowledgements

Would like to thank Anders Albrechtsen for valuable discussions. Furthermore, we would like to thank the Danish National Research Foundation (DNRF94), and the Danish Council for Independent Research (DFF-YDUN) for funding.

Supplementary Tables

AA	Aa	aa
$(f_l^A)^2$	$2f_l^A f_l^a$	$(f_l^a)^2$

Table S1: $P(G_l^i|f_l^A)$. The probability that the true genotype of individual i at locus l is G_l^i given that the population frequency of allele A at locus l is $f_l^A = 1 - f_l^a$.

G_l^i	G_l^j	X_l=0	X_l=1	X_l=2
AA	AA	$(f_l^A)^2$	f_l^A	1
AA	Aa	$2f_l^A f_l^a$	f_l^a	0
AA	aa	$(f_l^a)^2$	0	0
Aa	AA	$(f_l^A)^2$	$\frac{1}{2}f_l^A$	0
Aa	Aa	$2f_l^A f_l^a$	$\frac{1}{2}f_l^A + \frac{1}{2}f_l^a$	1
Aa	aa	$(f_l^a)^2$	$\frac{1}{2}f_l^a$	0
aa	AA	$(f_l^A)^2$	0	0
aa	Aa	$2f_l^A f_l^a$	f_l^A	0
aa	aa	$(f_l^a)^2$	f_l^a	1

Table S2: $P(G_l^j|f_l^A, X_l = m, G_l^i)$ for $m \in \{0, 1, 2\}$. The probability that the true genotype of individual j at locus l is G_l^j, given that at locus l the true genotype of individual i is G_l^i, that i and j share X_l alleles IBD and that the population frequency of allele A is $f_l^A = 1 - f^a$.

Pair	SNP chip based			NgsRelate			Genotype-based ML			PLINK		
	k_0	k_1	k_2	k_0	k_1	k_2	k_0	k_1	k_2	k_0	k_1	k_2
NA19470 NA19469	0.00	1.00	0.00	0.00	1.00	0.00	0.29	0.68	0.03	0.17	0.79	0.03
NA19381 NA19382	0.00	1.00	0.00	0.01	0.99	0.00	0.32	0.63	0.05	0.25	0.68	0.06
NA19470 NA19443	0.25	0.50	0.25	0.27	0.48	0.25	0.41	0.40	0.19	0.39	0.35	0.26
NA19443 NA19469	0.54	0.46	0.00	0.57	0.43	0.00	0.62	0.38	0.01	0.63	0.34	0.03
NA19380 NA19382	0.43	0.56	0.01	0.45	0.55	0.00	0.61	0.36	0.03	0.69	0.23	0.08
NA19380 NA19381	0.64	0.36	0.00	0.67	0.33	0.00	0.76	0.22	0.02	0.88	0.04	0.08

Table S3: Relatedness estimated for 6 pairs of related individuals based on real data using different estimation methods.

.

Supplementary Figures

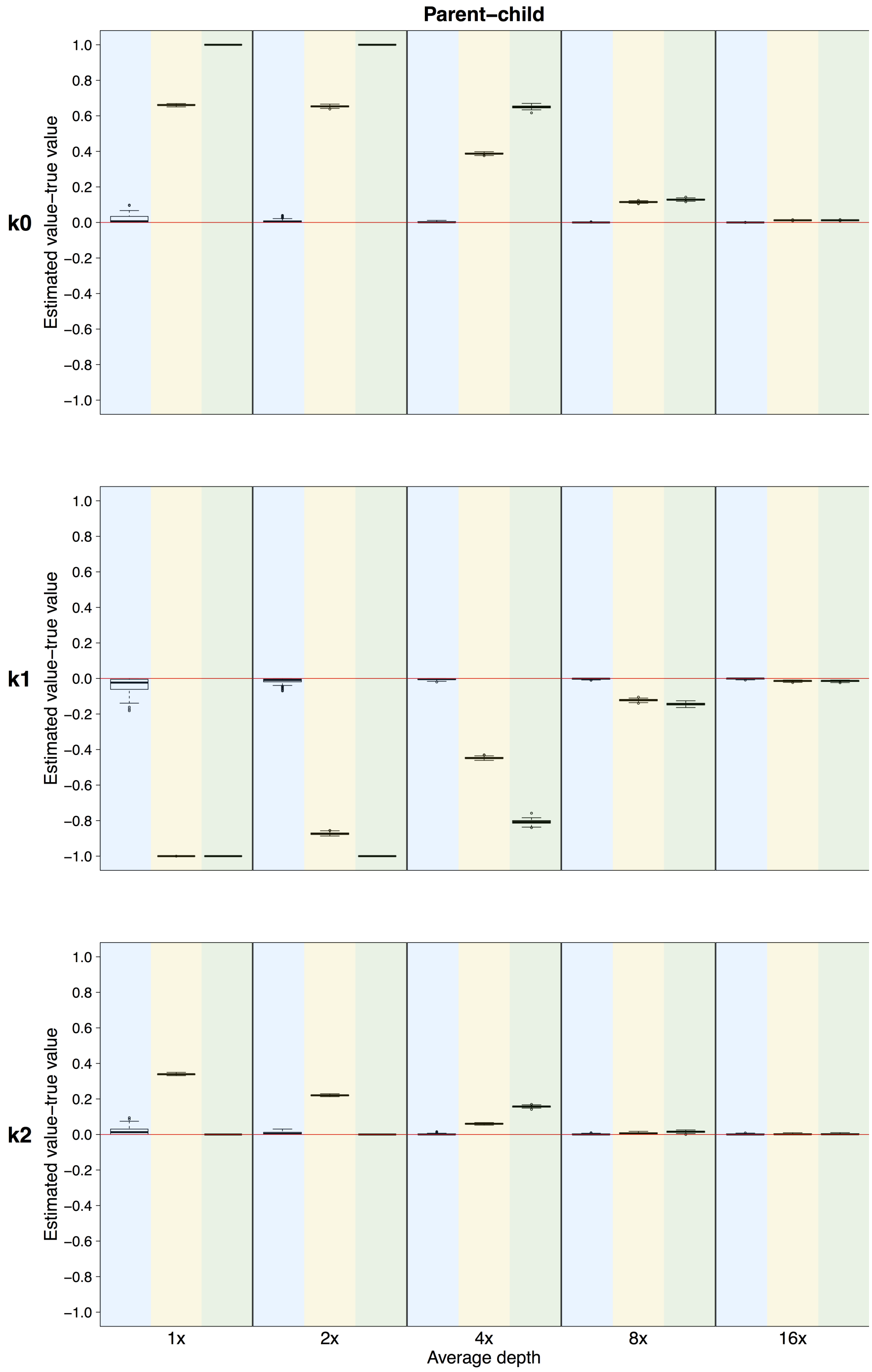

Figure S1: Difference between the estimated and the true $R = (k_0, k_1, k_2)$ for 100 parent-child pairs. The estimated values were obtained by applying NgsRelate (blue background), genotype based ML (yellow background) and PLINK (green background) to simulated data of five different average read depth; 1, 2, 4, 8 and 16. The horisontal red lines indicates the value expected when the estimated value is equal to the true value.

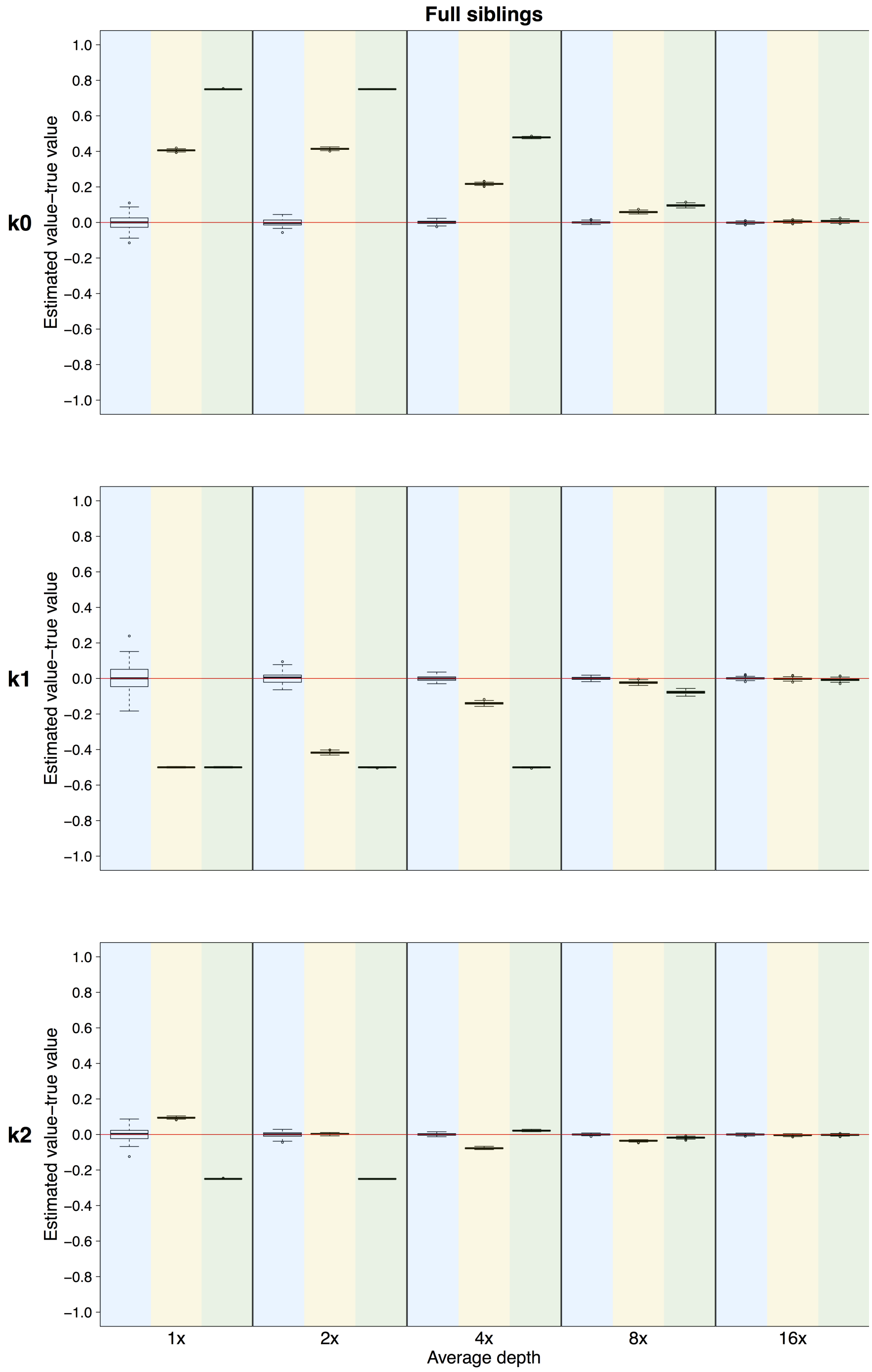

Figure S2: Difference between the estimated and the true $R = (k_0, k_1, k_2)$ for 100 full sibling pairs. The estimated values were obtained by applying NgsRelate (blue background), genotype based ML (yellow background) and PLINK (green background) to simulated data of five different average read depth; 1, 2, 4, 8 and 16. The horisontal red lines indicates the value expected when the estimated value is equal to the true value.

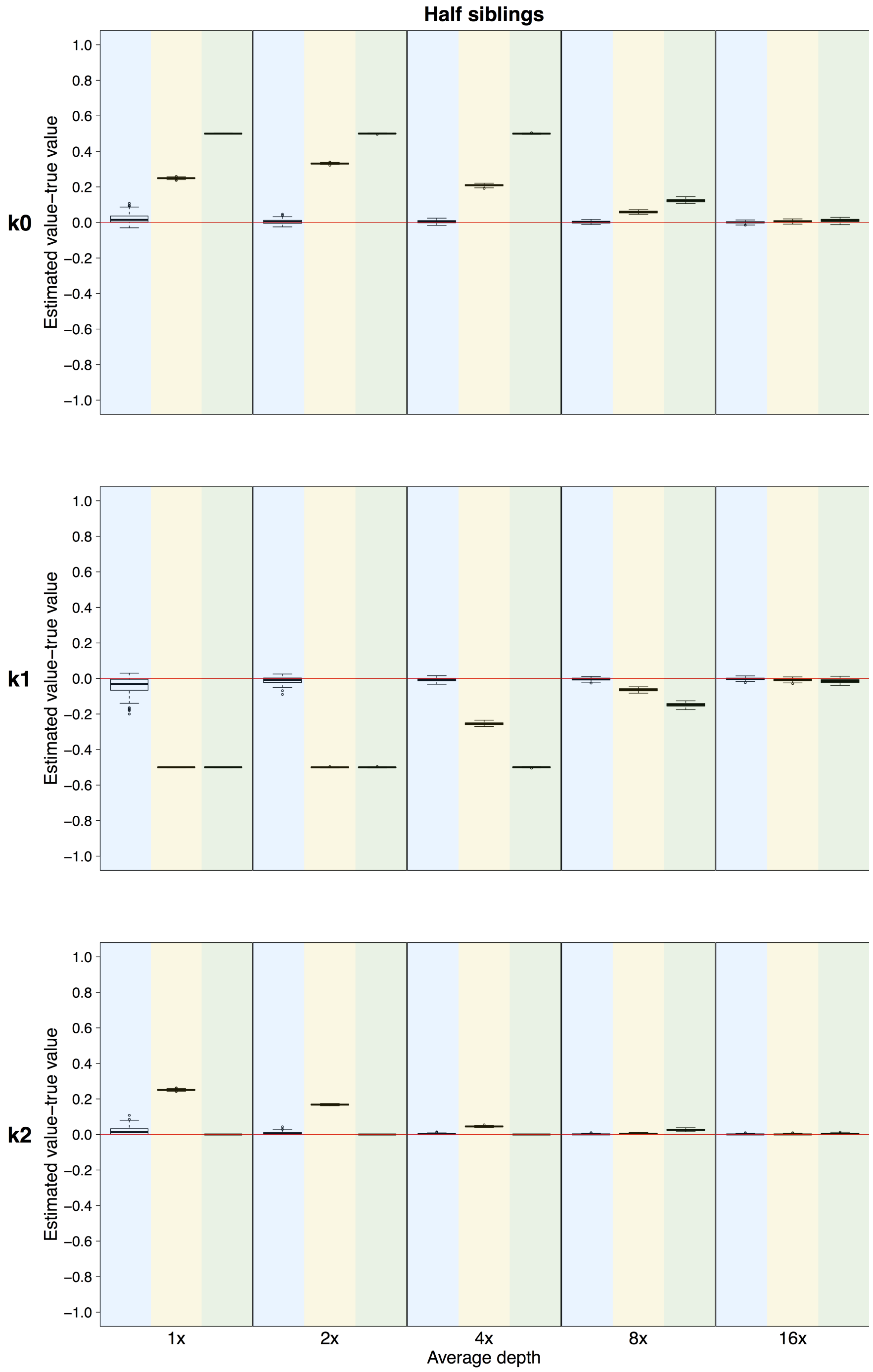

Figure S3: Difference between the estimated and the true $R = (k_0, k_1, k_2)$ for 100 half sibling pairs. The estimated values were obtained by applying NgsRelate (blue background), genotype based ML (yellow background) and PLINK (green background) to simulated data of five different average read depth; 1, 2, 4, 8 and 16. The horisontal red lines indicates the value expected when the estimated value is equal to the true value.

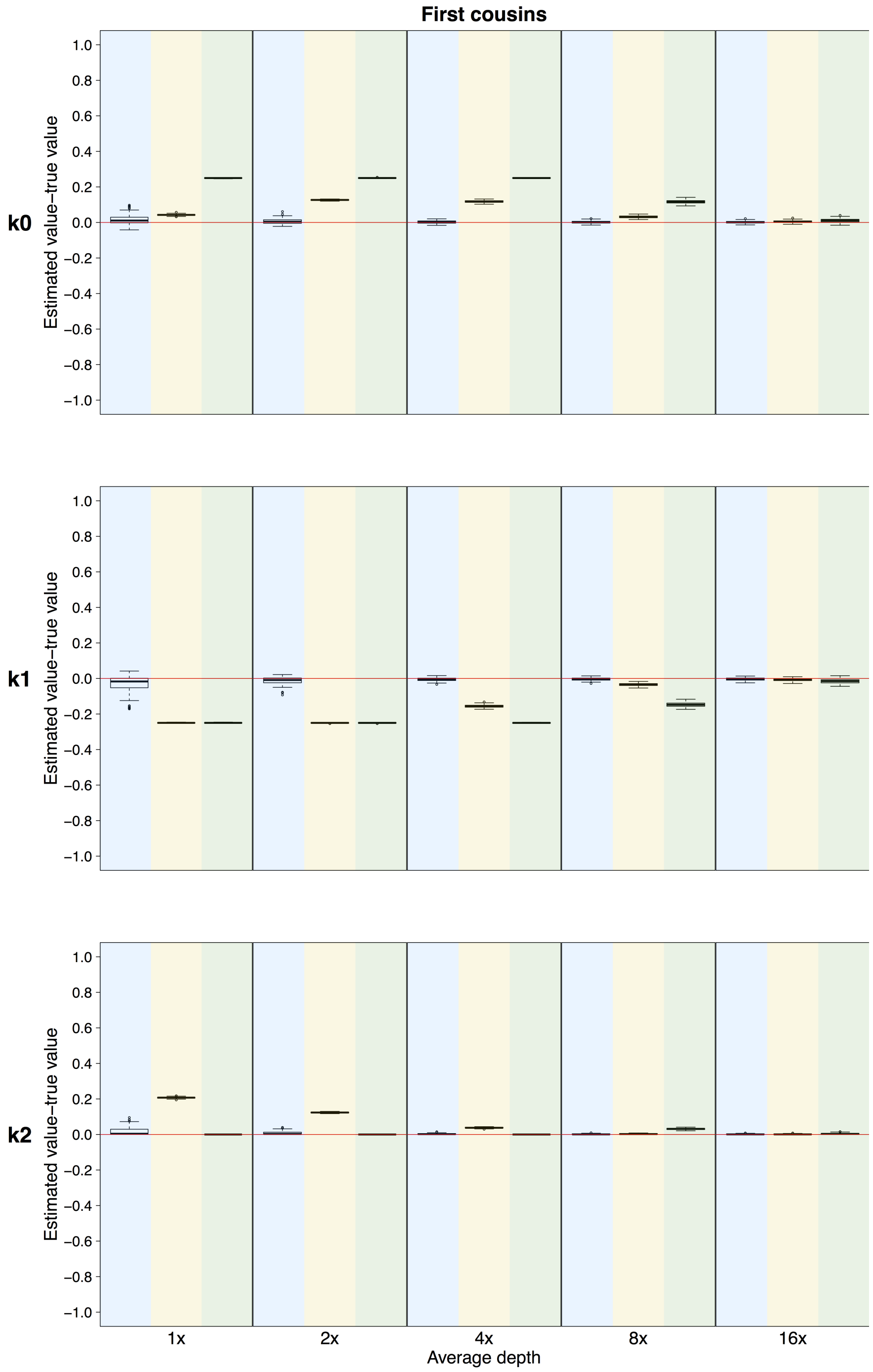

Figure S4: Difference between the estimated and the true $R = (k_0, k_1, k_2)$ for 100 first cousin pairs. The estimated values were obtained by applying NgsRelate (blue background), genotype based ML (yellow background) and PLINK (green background) to simulated data of five different average read depth; 1, 2, 4, 8 and 16. The horisontal red lines indicates the value expected when the estimated value is equal to the true value.

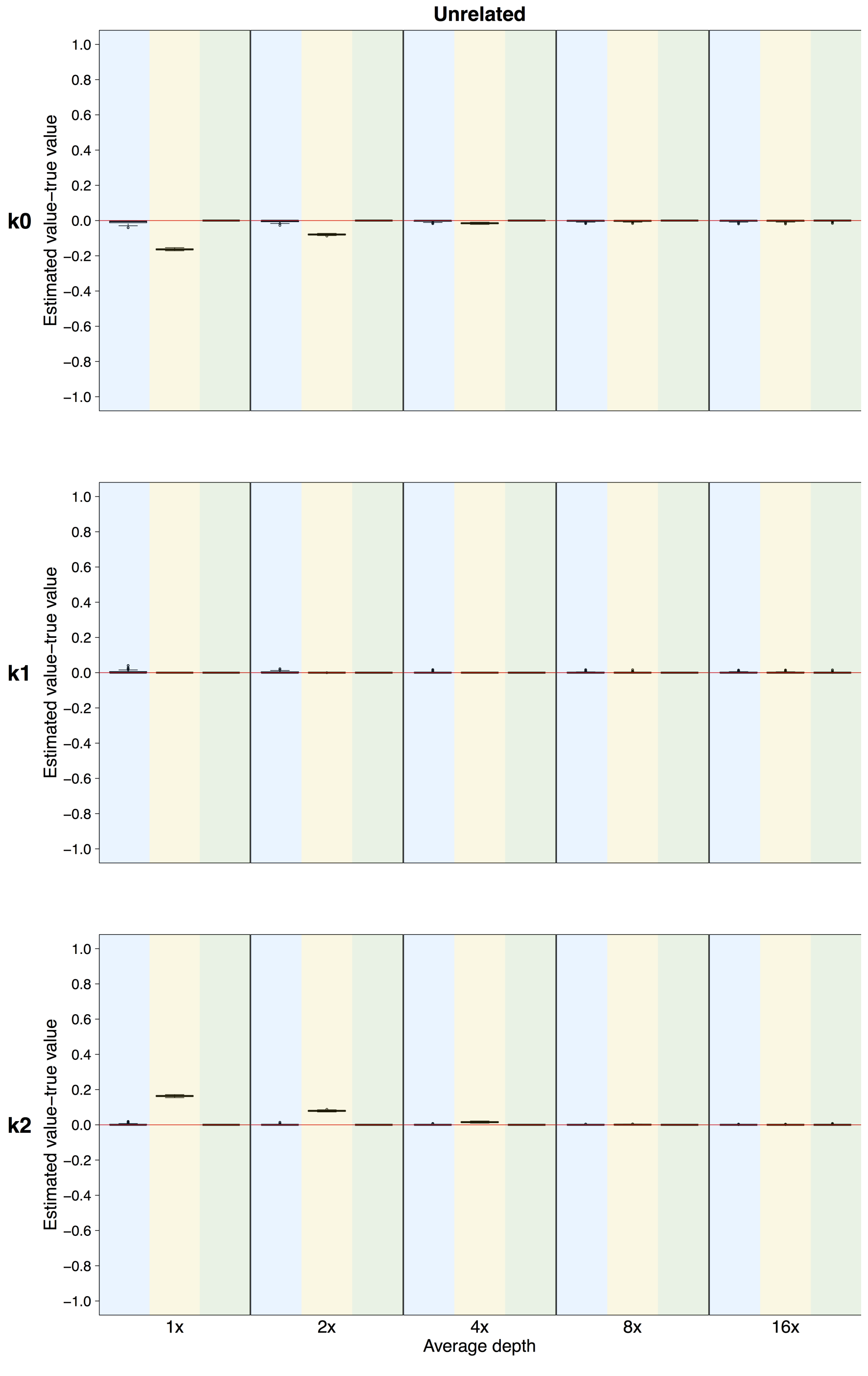

Figure S5: Difference between the estimated and the true $R = (k_0, k_1, k_2)$ for 100 unrelated pairs. The estimated values were obtained by applying NgsRelate (blue background), genotype based ML (yellow background) and PLINK (green background) to simulated data of five different average read depth; 1, 2, 4, 8 and 16. The horisontal red lines indicates the value expected when the estimated value is equal to the true value.

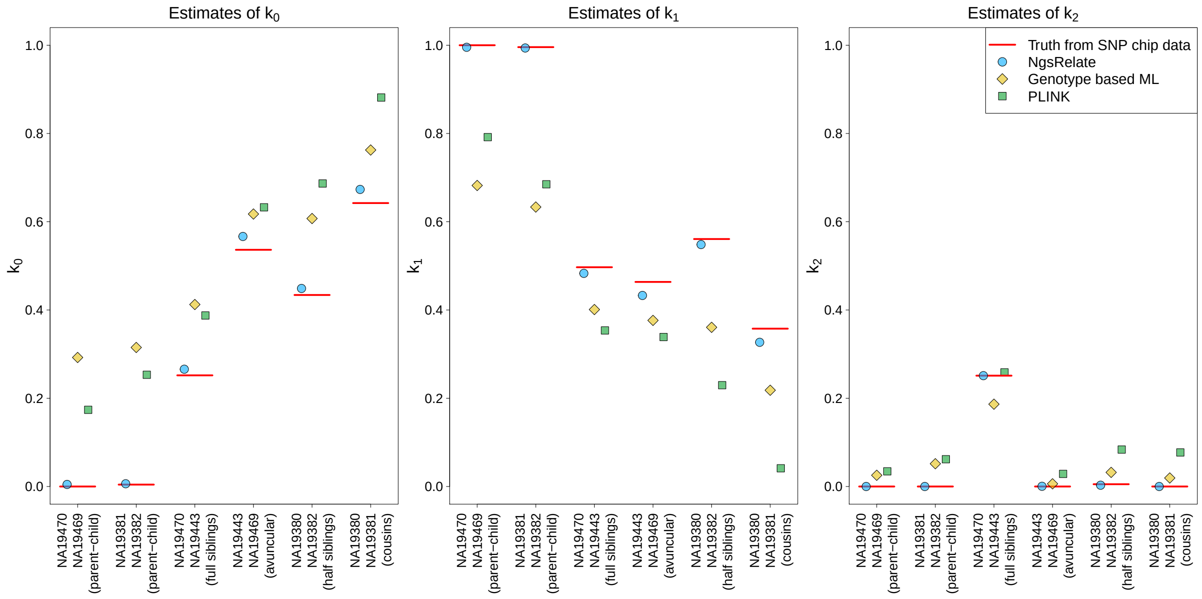

Figure S6: Estimated values of k_0, k_1 and k_2 for six related pairs of individuals. The colored symbols show the estimates obtained from low-depth NGS data from the 1000 Genomes Projects using three different methods. The red lines show the estimates obtained from high quality genotypes from SNP chips.

References

Choi, Y. *et al.*. Case-control association testing in the presence of unknown relationships. *Genet. Epidemiol.*, **33(8)**, 668–678.

International HapMap 3 Consortium *et al.* (2010). Integrating common and rare genetic variation in diverse human populations. *Nature*, **467**, 52–58.

Korneliussen, T. S. *et al.* (2014). ANGSD: Analysis of Next Generation Sequencing Data. *BMC Bioinformatics*, **15**, 356.

Li, H. (2011). Astatistical framework for SNP calling, mutation discovery, association mapping and population genetical parameter estimation from sequencing data. *Bioinformatics*, **27(21)**,2987–93.

McKenna, A. *et al.* (2010). The Genome Analysis Toolkit: a MapReduce framework for analyzing next-generation DNA sequencing data. *Genome Res.*, **20(9)**, 1297–303.

Purcell S. *et al.* (2007). PLINK: a toolset for whole-genome association and population-based linkage analysis. *Am. J. Hum. Genet.*, **81**, 559–75.

1000 Genomes Project Consortium *et al.* (2012). An integrated map of genetic variation from 1,092 human genomes. *Nature*, **491**, 56–65.

Varadhan, R. and Roland, C. (2008). Simple and globally convergent methods for accelerating the convergence of any EM algorithm. *Scand J Stat*, **35**, 335–353.
